# Ultrasound-guided microwave ablation for primary hyperparathyroidism in an elderly patient with pancytopenia: A case report

**DOI:** 10.1097/MD.0000000000043002

**Published:** 2025-07-11

**Authors:** Yun Cai, Yanwei Chen, Shuangshuang Zhao, Zheng Zhang, Huajiao Zhao, Feng Zhao, Baoding Chen

**Affiliations:** a Department of Ultrasound Medicine, Affiliated Hospital of Jiangsu University, Zhenjiang City, Jiangsu Province, China.

**Keywords:** contrast, elderly patients, enhanced ultrasound, microwave ablation, primary hyperparathyroidism

## Abstract

**Rationale:**

Primary hyperparathyroidism (PHPT) is the third major endocrine disorder characterized by increased secretion of parathyroid hormone, with an incidence of up to 3% in elderly women. Although current guidelines consider parathyroidectomy to be the only definitive treatment, it is extremely risky for elderly women with numerous underlying conditions. However, hypercalcemia and other clinical symptoms caused by PHPT can seriously threaten the health and quality of life of patients. Therefore, it is very important to find a safe and effective minimally invasive alternative therapy. Hence, we report a case of ultrasound-guided microwave ablation (MWA) for PHPT in an elderly patient with pancytopenia.

**Patient concerns:**

A 75-year-old female patient with pancytopenia was diagnosed with PHPT and was admitted for treatment due to her strong desire for treatment.

**Diagnoses:**

Imaging examination, laboratory examination and ultrasound-guided fine-needle aspiration cytology all led to a diagnosis of PHPT for the patient.

**Interventions:**

Considering the advanced age of the patient, suffering from pancytopenia and other underlying disease, the surgical risk is high, so MWA was selected for this hospitalization.

**Outcomes:**

We found that after 6 months of treatment, the following 4 indicators of the patient returned to normal (parathyroid hormone, 66.9 pg/mL; ionic calcium, 1.19 mmol/L; serum total calcium, 2.28 mmol/L; phosphorus, 0.80 mmol/L). The lesion volume of the patient gradually decreased, and the reduction rate of lesion volume reached 70.3% by 6 months after ablation. There were no complications, and no parathyroid nodules regenerated during follow-up.

**Lessons:**

Ultrasound-guided MWA represents a safe and effective therapeutic option for elderly patients with PHPT.

## 1. Introduction

Primary hyperparathyroidism (PHPT) is the third most common endocrine disease, following diabetes and thyroid disease, with its incidence steadily increasing each year.^[1]^ Statistics indicate that the prevalence of PHPT is higher in women than in men, reaching as high as 3% among women over 65 years of age.^[2]^ PHPT is usually caused by one or more parathyroid adenomas or glandular hyperplasia, with a single adenoma being the most common cause, accounting for about 75% to 85% of the total.^[3,4]^ PHPT is characterized by increased secretion of parathyroid hormone (PTH), which results in elevated serum calcium.^[5]^

PHPT can lead to a complex set of clinical symptoms. First, long-term PHPT leads to progressive loss of bone calcium with secondary osteopenia and kidney damage, leading to osteoporosis and fragility fractures.^[2,6]^ Secondly, chronic hypercalcemia may contribute to a heightened risk of calcified plaques, which raises the incidence of vaso-occlusive events and infarctions.^[7]^ In addition, several reports have suggested that PHPT may increase the risk of acute pancreatitis.^[8,9]^ All these undoubtedly increase the mortality of elderly patients with PHPT. Parathyroidectomy is currently the preferred treatment for PHPT, but it is necessary to find a minimally invasive and safe treatment for older adults with underlying conditions that do not meet surgical indications.^[10]^ In recent years, microwave ablation (MWA) has been successfully applied to the treatment of benign and malignant tumors in thyroid, liver, and other organs because it has the advantages of high safety, small invasion, and high repeatability.^[11,12]^ Up to now, few cases have been reported on MWA for elderly patients with PHPT. Here, we report a case of ultrasound-guided percutaneous MWA of parathyroid adenoma in a 75-year-old woman with pancytopenia.

### 1.1. Consent

This study was approved by the ethics committee of Jiangsu University (approval no: KY2024H0516-01). This study did not involve vulnerable/minor population. The patient signed an informed consent form agreeing to publish this case report and any associated images.

## 2. Case report

**Figure 1.**
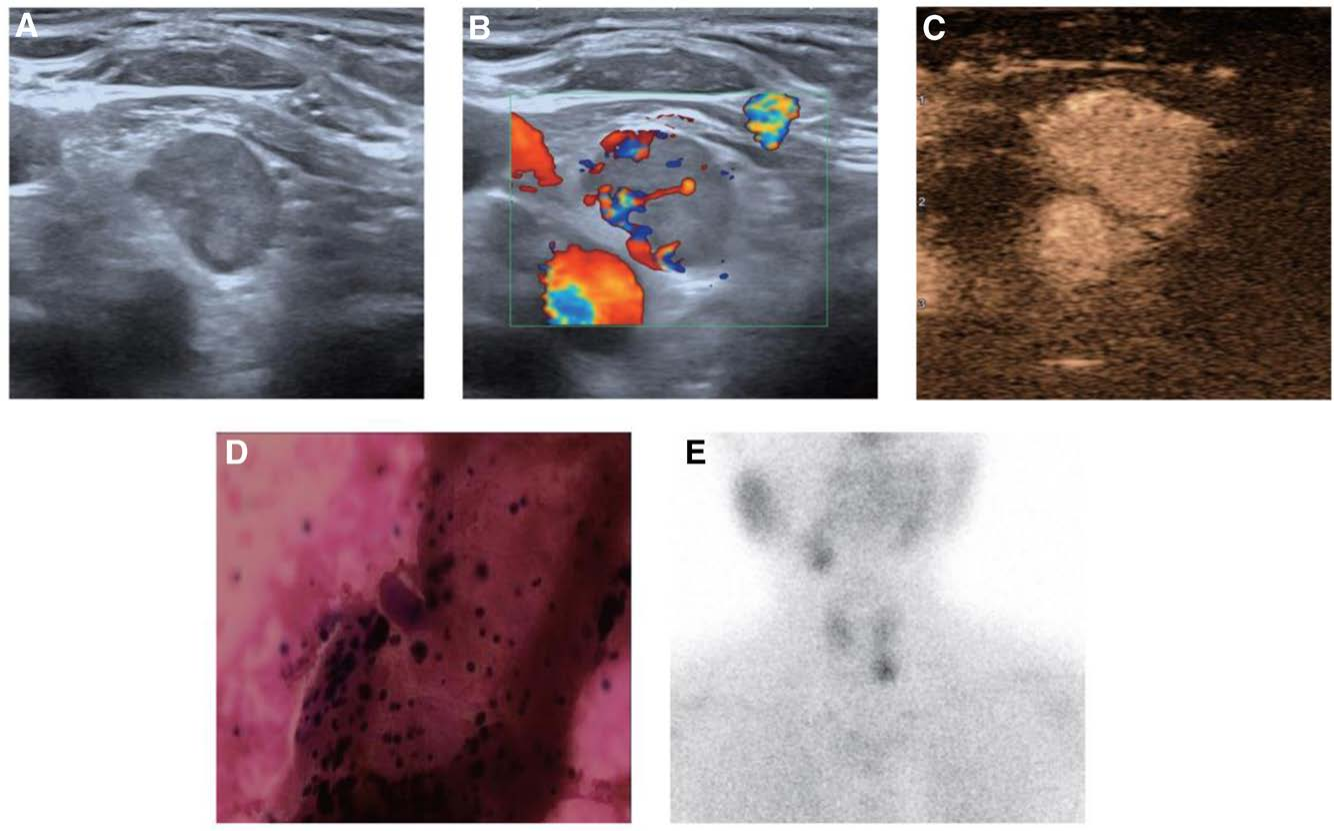
Preoperative examinations of the patient. (A) Conventional 2-dimensional ultrasound showed a low echo nodule on the dorsal side of the left inferior lobe of the thyroid, with clear boundary and irregular shape. (B) Color Doppler flow imaging (CDFI) showed that abundant blood flow signals could be detected inside and around the lesion. (C) Contrast-enhanced ultrasound (CEUS) showed that the lesion was uniformly enhanced during the arterial phase. (D) Pathology revealed that there were a small number of follicular epithelioid cells, and parathyroid cells were not excluded. (E) 99mTc-ses-tamibi SPECT showed localized nuclide concentration shadow at the lower pole of the left lobe of the thyroid gland. CEUS = contrast-enhanced ultrasound, CDFI = color Doppler flow imaging, SPECT = single photon emission computed tomography.

A 75-year-old female was suspected of having a thyroid nodule during physical examination 1 year prior, so she came to our hospital for further diagnosis and treatment. Ultrasound examination showed that there was a low echo nodule on the dorsal side of the left inferior lobe of the thyroid, approximately 11 × 10 × 18 mm, with clear boundary and irregular shape (Fig. 1A). Color Doppler flow imaging showed that abundant blood flow signals could be detected inside and around the lesion (Fig. 1B). Contrast-enhanced ultrasound (CEUS) showed that the lesion was uniformly enhanced during the arterial phase (Fig. 1C). Then ultrasound-guided fine-needle aspiration cytology was performed, and pathology revealed that there were a small number of follicular epithelioid cells, and parathyroid cells were not excluded (Fig. 1D). The preliminary laboratory results were as follows: PTH, 258.1 pg/mL; ionized calcium, 1.36 mmol/L; serum total calcium, 2.62 mmol/L; phosphorus, 0.72 mmol/L; 25-hydroxyvitamin D, 9.53 ng/mL. And 99mTc-ses-tamibi single photon emission computed tomography showed localized nuclide concentration shadow at the lower pole of the left lobe of the thyroid gland which was considered hyperfunctional parathyroid tissue imaging (Fig. 1E). Combined with the above laboratory and imaging examinations, the patient was preliminarily diagnosed as PHPT.

Due to the patient had a strong desire for treatment, she was admitted to our hospital. The results of the biochemical examination after admission were as follow: pancytopenia (red blood cell count, 3.15 × 10^12^/L; white blood cell count, 2.0 × 10^9^/L; platelet count, 70 × 10^9^/L); abnormal coagulation function (d-dimer, 1.00 mg/L); abnormal liver and kidney function (albumin, 35.9 g/L; total bilirubin, 31.6 μmol/L; glucose, 7.61 mmol/L, etc.); abnormal tumor marker (carbohydrate antigen 125, 65.90 U/mL). Considering the patient’s advanced age ansd the serious underlying diseases of the patient, the risk of surgical operation was high. After multidisciplinary team consultation, it was finally decided to use ultrasound-guided percutaneous MWA for the treatment.

**Figure 2.**
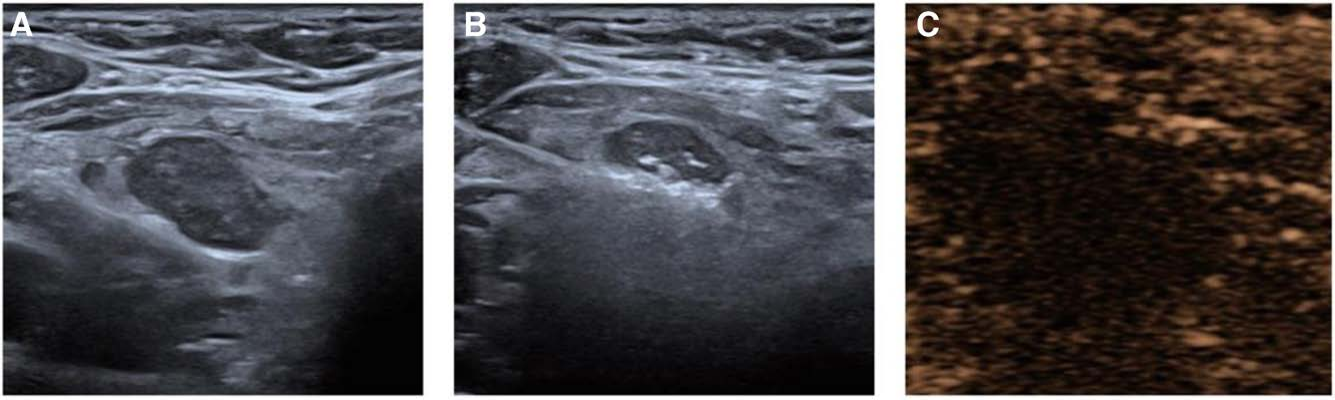
Ultrasound images of patients during ablation. (A) Normal saline was injected between the parathyroid lesion and important surrounding structures to form a fluid isolation area. (B) Ultrasound-guided microwave ablation of parathyroid nodule. (C) Contrast-enhanced ultrasound was performed immediately after the operation, showing no enhancement within the lesion, which proved that the ablation was complete.

The patient performed platelet transfusion 2 days before surgery to improve her coagulation function. After the patient’s coagulation function met the requirements of the operation, the operation was performed. During the surgery, the patient was placed in the supine position with the neck fully exposed. The surgical site was routinely disinfected and covered with sterile towels. According to the location of the lesion, 1% lidocaine was used for local infiltration anesthesia. Under ultrasound guidance, 0.9% normal saline was injected between the parathyroid lesion and important surrounding structures such as trachea, esophagus, and recurrent laryngeal nerve to form a fluid isolation area with a width of >5 mm (Fig. 2A). Then, the MWA needle was inserted into the lesion, and the ablation was performed point by point and region by region by continuous moving method until the ablation point covered the entire lesion (Fig. 2B). When the ablation needle was removed after ablation, the needle path was also ablated to prevent needle planting. The ablation power was 30 W and the ablation time was 7 minutes and 41 seconds. CEUS was performed immediately after the operation, showing no enhancement within the lesion, which proved that the ablation was complete (Fig. 2C). The operation went smoothly. PTH, ionic calcium, serum total calcium, and phosphorus were all returned to the normal range 1 day after surgery, and no related complications occurred. The patient was discharged satisfactorily.

Then, we followed up the patient with laboratory serum biochemical indicators and ultrasound at 1 day, 3 days, 1 month, 3 months, and 6 months after ablation. We found that after 6 months of treatment, the following 4 indicators of the patient returned to normal (PTH, 66.9 pg/mL; ionic calcium, 1.19 mmol/L; serum total calcium, 2.28 mmol/L; phosphorus, 0.80 mmol/L; Table 1). The lesion volume of the patient gradually decreased, and the reduction rate of lesion volume reached 70.3% by 6 months after ablation (Table 1). Importantly, there were no complications, and no parathyroid nodules regenerated during the follow-up. According to the criteria for surgical resection (PTH and serum calcium returned to normal at 6 months postoperatively), the patient met the criteria for cure.^[13]^

**Table 1:** Serum biochemical data and ultrasound data of the patient during follow up.

Follow-up time	PTH (pg/mL)	Ionic calcium (mmol/L)	Serum total calcium (mmol/L)	Phosphorus (mmol/L)	Volume (cm^3^)	VRR (%)
Before MWA	258.1	1.36	2.62	0.72	0.91	–
1 D post-MWA	73.2	1.27	2.30	0.91	0.66	27.4
3 D post-MWA	102.8	1.18	2.04	0.93	0.41	54.9
1 M post-MWA	89.0	1.03	2.23	0.99	0.36	60.4
3 M post-MWA	65.0	1.09	2.21	0.92	0.35	61.5
6 M post-MWA	66.9	1.19	2.28	0.80	0.27	70.3

Normal range: PTH, 12–88 pg/mL; ionic calcium, 0.95–1.35 mmol/L; serum total calcium, 2.08–2.60 mmol/L phosphorus, 0.86–1.51 mmol/L.

D = day, M = month, MWA = microwave ablation, PTH = parathyroid hormone, VRR = volume reduction rate.

## 3. Discussion

PHPT is the most common cause of chronic hypercalcemia.^[7]^ Its clinical symptoms are relatively concealed and varied, such as skin pruritus, nephrolithiasis, and fracture.^[14]^ Even though approximately 80% of asymptomatic patients do not have these symptoms, it can invisibly accelerate bone turnover, increasing the risk of ostealgia and fragility fractures.^[13,15]^ Morbidity and mortality from these fractures are more common in elderly patients. Surgery remains the only definitive treatment for PHPT. However, for the elderly, advanced age and physiological comorbidity might increase the risk of surgery, so most of them lose or forgo the opportunity of surgery, thus missing the opportunity to restore BMD, prevent fractures and improve quality of life. MWA offers distinct advantages including high safety, minimal invasiveness, and a brief treatment duration, thereby making it an excellent alternative therapeutic option for elderly patients.

In recent years, CEUS has emerged as a critical tool for both preoperative localization and postoperative evaluation. This is attributed to its advantages in enhancing spatial resolution, precisely delineating lesions, and accurately differentiating surrounding tissues.^[16]^ Given that hyperfunctional parathyroid lesions usually have an abundant blood supply, CEUS can dynamically detect microvascular perfusion which can be considered a reliable sign of preoperative localization of parathyroid adenomas.^[17]^ After ablation, CEUS can be performed to evaluate the ablation area and determine whether the ablation is complete. If there is still contrast filling in the lesion after ablation, additional ablation can be performed again until the ablation is complete. This can greatly improve cure rates and optimize treatment outcomes.^[18]^ With the aid of CEUS, our patient had a successful operation.

It has been reported in the literature that the parathyroid cells overflowed during surgery or fine needle puncture which resulted in recurrent hyperparathyroidism because parathyroid cells can regenerate and duplicate.^[19,20]^ In this case, we adopted the method of ablating the needle path after ablation to reduce the possibility of needle implantation and avoid the risk of postoperative recurrence.

We performed ultrasound-guided MWA on an elderly patient, taking into account factors such as advanced age, underlying diseases, and pancytopenia. The serum PTH, calcium, phosphorus of this patient significantly improved without any complications and reached the cure standard after 6 months after MWA. However, this patient was followed up for a short period of time, and we will continue to follow up in the future to evaluate the long-term outcome.

## 4. Conclusion

In conclusion, we successfully utilized MWA to manage an elderly patient with PHPT and pancytopenia, thereby alleviating the long-term health risks associated with hypercalcemia. Therefore, ultrasound-guided MWA represents a safe and effective therapeutic option for elderly patients with PHPT, potentially serving as a viable alternative to parathyroidectomy.

## Author contributions

**Conceptualization:** Shuangshuang Zhao.

**Data curation:** Yanwei Chen.

**Formal analysis:** Yun Cai, Yanwei Chen, Zheng Zhang, Huajiao Zhao.

**Funding acquisition:** Baoding Chen.

**Methodology:** Shuangshuang Zhao.

**Writing – original draft:** Yun Cai.

**Writing – review & editing:** Feng Zhao, Baoding Chen.
